# Estimating the range of incremental cost-effectiveness thresholds for healthcare based on willingness to pay and GDP per capita: A systematic review

**DOI:** 10.1371/journal.pone.0266934

**Published:** 2022-04-14

**Authors:** Haru Iino, Masayuki Hashiguchi, Satoko Hori

**Affiliations:** Division of Drug Informatics, Keio University Faculty of Pharmacy, Tokyo, Japan; Xiamen University, CHINA

## Abstract

**Background:**

Decision-making in healthcare policy involves assessing both costs and benefits. In determining the cost-effectiveness (CE) threshold, willingness to pay (WTP) per quality-adjusted life year (QALY), GDP per capita, and other factors are important. However, the relationship between WTP/QALY or GDP per capita and the CE threshold is unclear. It is important to clarify the relationship between WTP/QALY and GDP to provide a clear basis for setting the CE threshold.

**Objective:**

The purpose of this study was to compare WTP/QALY and GDP per capita, and to develop a new CE threshold range based on WTP using GDP per capita. The relationship between WTP/QALY and healthy life expectancy (HALE) was also investigated.

**Methods:**

We searched MEDLINE, EMBASE and Web of Science from 1980/01/01 to 2020/12/31 using the following selection criteria (latest search: Dec 2021):1, studies that estimated WTP/QALY; 2, the general population was surveyed; 3, the article was in English. From the collected articles, we obtained average values of WTP/QALY for various countries and compared WTP/QALY with GDP per capita. The correlation between WTP/QALY and HALE was also examined.

**Results:**

We identified 20 papers from 17 countries. Comparison of mean WTP/QALY values with GDP per capita showed that most WTP/QALY values were in the range of 0.5–1.5 times GDP per capita, though the median values were less than 0.5 times. Comparison of WTP/QALY with HALE showed a statistically significant positive correlation when Taiwan was excluded as an outlier.

**Conclusions:**

Our results suggest a CE threshold range of 0.5–1.5 times GDP per capita is appropriate but lower than the WHO-recommended range of 1–3 times. The correlation between WTP/QALY and HALE suggests that investment in healthcare is reflected in an increased healthy life expectancy. Since WTP is based on consumer preferences, this range could be used to set a generally acceptable criterion.

## Introduction

Improvements in medical technology and sanitation have significantly prolonged human life expectancy. However, the development of new health technologies is expensive, and the cost of pharmaceuticals is also increasing. As a result, health care spending as a percentage of gross domestic product (GDP) has been increasing globally, and there is a debate as to how available funds should best be spent [1]. Health technology assessment (HTA) has been used to consider both effectiveness and cost, since medical resources are finite and, in many cases, publicly financed through taxation [2].

Cost-effectiveness analysis in HTA is conducted by calculating the incremental cost-effectiveness ratio and determining cost-effectiveness based on the cost-effectiveness threshold (CE threshold) [3]. In particular, when determining the threshold for insurance reimbursement, GDP per capita or willingness to pay per quality-adjusted life year (WTP/QALY), which can be used to calculate the cost of general health conditions. This may be used as a reference to set the same threshold for all items rather than for specific diseases. The CE threshold has an important role in HTA, as it can be used to determine whether reimbursement is appropriate.

The World Health Organization (WHO) recommends a threshold of one to three times the GDP per capita for the cost of investing in one disability-adjusted life year (DALY), which is widely known and often cited when discussing CE thresholds. However, the rationale for the upper limit of three times is not well defined, and is insufficient as a basis for decision-making [4,5]. Since the rationale for a criterion based on GDP per capita is unclear, Wood et al. (2016) estimated a range of thresholds based on opportunity cost, resulting in a range of 1–51% of GDP per capita for low- and middle-income countries and 18–71% for middle- and high-income countries [5].

One way to determine the C/E threshold is to use the opportunity cost approach; that is, the value of health that would have to be given up by investing the healthcare budget in a healthcare service, or to measure the willingness to pay (WTP) for a year of staying healthy [6]. The setting of thresholds can affect access to health care and the use of public funds. For this reason, we think it is better to reflect people’s intentions/preferences in setting the threshold. The WTP approach measures people’s preferences, so that by focusing on this approach, we may find evidence for setting a threshold that is more generally acceptable to people.

In WTP-based methods, the WTP for one quality adjusted life year (QALY) is usually used. In addition, the threshold is often set by referring to surveys of the general health status for a population, rather than specific health conditions, and where systematic reviews of these surveys have been reported [7–10]. From these studies, it is known that WTP is affected by many factors, including survey methods, survey population, and calculation methods, but few studies have examined the relationship between GDP per capita and WTP/QALY. Furthermore, even in those studies, there are large differences in study design, for example whether the survey is based on stated or revealed preferences, and also in evaluation, and thus the quality of the results is insufficient [7,9].

Therefore, the purpose of this study was to clarify the appropriate range of GDP per capita based on WTP/QALY values by organizing the available data using our criteria. In this study, we will not only summarize the ratio of WTP/QALY to GDP per capita, but also identify the range within which most WTP/QALY values fit. That range can be used as a guide for threshold setting even in countries that have not conducted WTP surveys before. Also, since this range is based on consumer preferences, it should be possible to set a threshold that the general population would regard as acceptable.

We also examined the correlation between Healthy Life Expectancy (HALE) and WTP/QALY to investigate whether there is a relationship between WTP/QALY and people’s health status. HALE is the value of life expectancy weighted by health status. It indicates the expected life expectancy assuming overall survival in a completely health state.

In this article, we often compare cost/DALY and cost/QALY. The WHO criteria are based on DALYs, while the values calculated by WTP and many health economic studies are based on QALYs. Although they are different in calculations and definitions, their meaning is the same: survival in a healthy state [11]. Cost/QALY and Cost/DALY both indicate the cost of gaining one perfectly healthy life year and can therefore be validly compared [12].

## Methods

### Article search

**Fig 1:**
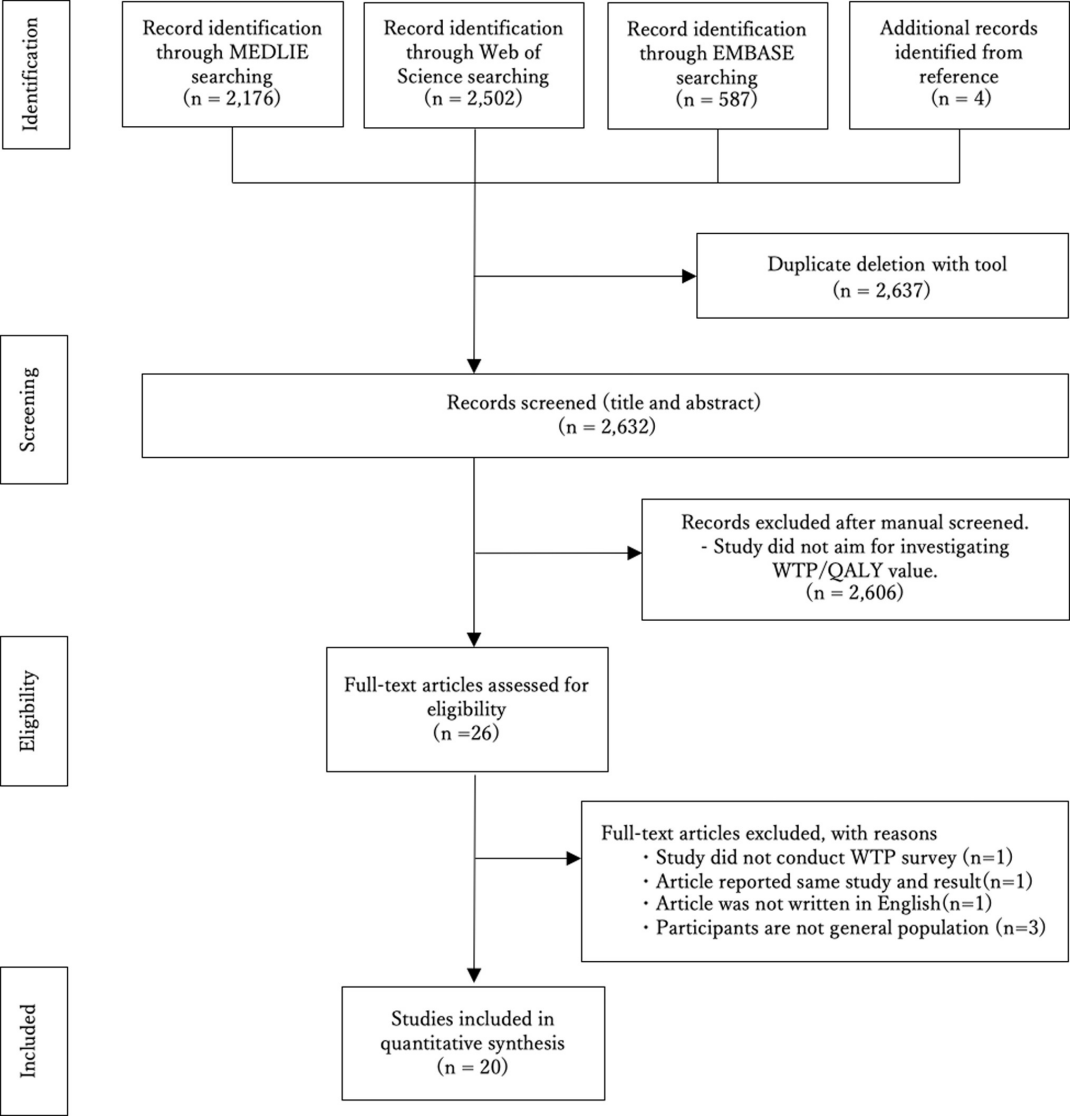
Flow diagram of study selection.

We conducted a systematic search using MEDLINE, EMBASE and Web of Science. The search terms used were "quality adjusted life year" "quality adjusted life years" "disability adjusted life year" "disability adjusted life years" "cost benefit analysis" and "willingness to pay" and the search period was specified as between 1980/01/01 and 2020/12/31 based on publication date. For instance, the detailed search in MEDLINE is ("quality adjusted life year"[All Fields] OR "quality adjusted life years"[All Fields] OR "disability adjusted life year"[All Fields] OR "disability adjusted life years"[All Fields]) AND "cost benefit analysis"[All Fields] AND "willingness to pay"[All Fields] AND 1980/01/01:2020/12/31[Date—Publication]. Articles retrieved were reviewed based on the following criteria: 1, studies that estimated WTP/QALY; 2, the general population was surveyed; 3, the article was in English. Database searches were conducted from the start of the research until December 2021. The search, removal of duplicates, and initial screening were done by a single author (HI). Screening of potentially compatible references was done independently by two authors (HI, HM). If there was any disagreement, the third author (SH) provided advice. A flow diagram of the selection procedure is shown in Fig 1. The searching process is shown in detail in Supporting information (S1 File). Management of literature and removal of duplicates were done by Mendeley Desktop Version 1.19.8 (Mendeley Ltd).

### Data collection and extraction

The value of statistical life (VSL) method shows revealed preference against the risk of death, and the calculated value is higher than values obtained using other methods [8,13]. There are also some reports that values calculated using the visual analog scale (VAS) have a high ceiling effect, and that the correlation with health status is low to begin with, so that reliability is questionable. Therefore, in this study, we excluded VSL values and values measured using VAS only [14–16]. However, we did not exclude the case where VAS was used in conjunction with EQ-5D.

### Data integration, analysis

WTP/QALY values were converted to international dollars in 2019 using purchasing power parity (PPP) and the inflation rate; PPP values were taken from the International Monetary Fund database, and the US Inflation Calculator was used to adjust for inflation [17,18]. WTP/QALY was calculated using Euro, but where the country’s currency was not Euro, it has been converted using PPP value as EU. The EU PPP was taken from the Organization for Economic Co-operation and Development (OECD) [19]. All the conversion rates are survey year values. If the survey year was unknown, the publication year of the report was used.

When the mean and median of WTP/QALYs were reported together in an article, they were analyzed separately. In cases where there were multiple mean and median values for a country, the respective averages were calculated and used as representative values for that country. (For example, suppose that for one country there are three means [1000, 2500, 2500] and two medians [1000, 2000] available; in that case, the country would have a mean of 2000 and a median of 1500.) Where a unique calculated value of WTP/QALY that is neither the mean nor the median was shown, it was treated as being equivalent to the mean value. In cases where WTP/QALYs were calculated for two populations (patients and general people), only the results for the general people were used, not the results for patients.

For comparison between GDP per capita and WTP/QALY, we plotted the respective values and drew lines representing one and three times GDP per capita, the range of threshold values recommended by the WHO [4]. Then, *n* times lines were extrapolated such that the WTP/QALY values for countries were fitted appropriately. Correlation analysis was performed for WTP/QALY and HALE, and GDP per capita was taken from the International Monetary Fund database as converted to international dollars according to PPP [20]. HALE data were taken from the WHO website, except for Taiwan, where the average for men and women was obtained from the Global Burden of Disease Study 2017 [21,22]. In addition, we cite the GNI-based classification presented by the World Bank as a tool for classifying countries [23]. Correlation analysis was performed using IBM SPSS Statistics for Mac ver26 (IBM SPSS Inc., Chicago, USA). Cook’s distance and DFFITS were calculated as statistical diagnostics for outliers in the regression. Cook’s distance and DFFITS were calculated using Python 3.7 and its module statsmodels v0.13.1[24,25]. Analysis of the data was performed by one author (IH) and the methods and results were reviewed independently by two authors (MH, SH). This review was not registered in the registry. Also, the protocol was not prepared.

## Results

The search (search date: March 2021) yielded 20 articles that met the final selection criteria (Fig 1). From the articles, we obtained 233 data on the mean WTP/QALY for 17 countries, including 106 median WTP/QALYs in 16 countries. The obtained articles and WTP/QALY values are summarized in Table 1. All the collected and processed data are shown in the Supporting Information (S1 Table, S1 Fig). Some examples of studies that were excluded by screening are as follows. A study by LauraVallejo-Torres (2016) calculated WTP/QALYs, but the values were calculated using a historical database and did not actually investigate WTP [26]. The study by Jesus Martín-Fernández (2014) targets patients rather than the general population [27]. Such studies do not fit the criteria for adoption and are therefore not included in this study.

**Table 1:** List of WTP/QALY values from the literature [28–46].

Country	Author	Survey year	Sample size (total respondents on the article)	perspective	Scenario type	QALY measuring method	Number of WTP/QALY mean estimates (median)	Currency unit	Range of WTP/QALY mean (median)	average of WTP/QALY	WTP/QALY mean USD2019 adjusted Value (median)	Average of WTP/QALY mean USD2019 Value (median)	GNI income categories
**Australia**	Shiroiwa et al. (2010) [28]	2008	1000 (5620)	Individual and Societal	General health	-	3(1)	AUD	64000–89,000 (36,000)	77000 (36000)	62198 (30,146)	62198 (30,146)	High-income
**China**	Zhao et al. (2011) [29]	2009	632	Individual and Societal	Chronic prostatitis and General health (Own current health status)	EQ-5D, SF-6D	2	USD	4711–5,012	4,862	5,525	5,525	Upper-middle-income
**Denmark**	Gyld-hansen et al. (2003) [30]	2001	3201	Individual	General health	EQ-5D	2	DKK	74109–88,000	81,055	15,820	33695 (12,248)	High-income
	Gyld-hansen et al. (2012) [16]	N/A	1724	Individual	General health	EQ-5D, TTO	6(2)	EUR	2740–44,565 (60770–92,307)	22571 (76539)	31176 (11,034)		
	Robinson et al. (2013) [31]	2008	2637 (21896)	Individual	General health	EQ-5D, TTO, SG	8(8)	EUR	24796–57,389 (5749–15,409)	36722 (9,257)	54089 (13,462)		
**France**	Robinson et al. (2013) [31]	2008	2674 (21896)	Individual	General health	EQ-5D, TTO, SG	8(8)	EUR	11317–26,890 (2745–4,574)	19954 (3,520)	29251 (4,832)	29251 (4,832)	High-income
**Greece**	Afentoula M et al. (2020) [32]	2019	528	Individual	General health	EQ-5D	5(5)	EUR	11176–27487 (2167–6070)	19952 (3222)	36276 (5859)	36276 (5859)	High-income
**Hungary**	Robinson et al. (2013) [31]	2008	2287 (21896)	Individual	General health	EQ-5D, TTO, SG	8(8)	EUR	10938–26,132 (3081–7,671)	17945 (4,975)	26432 (7,235)	26432 (7,235)	High-income
**Japan**	Shiroiwa et al. (2010) [28]	2008	1114 (5620)	Individual and Societal	General health	-	3(1)	JPY	5000000–6,400,000 (3,100,000)	5600000 (3,100,000)	57009 (31,559)	55578 (44,194)	High-income
	Shiroiwa et al. (2013) [33]	2011	2283	Individual	General health	EQ-5D	1(1)	USD	50000 (50,000)	50000 (50,000)	56828 (56,828)		
	Igarashi et al. (2019) [34]	N/A	1000	Individual and Societal	General health	EQ-5D	9	JPY	2600000–14,900,000	5,374,444	52,898		
**Netherland**	Bobinac et al. (2010) [35]	2008	1091	Individual	General health	EQ-5D	1	EUR	24,500	24,500	34,065	56624 (25,962)	High-income
	Bobinac et al. (2012) [36]	N/A	1004	Societal	General health	EQ-5D	4	EUR	52200–83,200	64,925	88,164		
	Bobinac et al. (2013) [37]	N/A	1004	Individual	General health	EQ-5D	6(3)	EUR	55400–113,200 (23500–37,900)	85567 (32,767)	117380 (44,949)		
	Bobinac et al. (2013) [38]	2010	1091	Individual	General health	EQ-5D	29	EUR	1200–21,400	9,162	12,637		
	Robinson et al. (2013) [31]	2008	2510 (21896)	Individual	General health	EQ-5D, TTO, SG	8(8)	EUR	15738–27,418 (3415–7,904)	22022 (5,039)	30873 (6,975)		
**Norway**	Robinson et al. (2013) [31]	2008	2020 (21896)	Individual	General health	EQ-5D, TTO, SG	8(8)	EUR	21602–41,298 (7621–15,472)	31956 (9,389)	47068 (13,654)	47068 (13,654)	High-income
**Poland**	Robinson et al. (2013) [31]	2008	2173 (21896)	Individual	General health	EQ-5D, TTO, SG	8(8)	EUR	18601–40,023 (3611–10,748)	28368 (6,788)	41784 (9,872)	41784 (9,872)	High-income
**Republic of Korea**	Shiroiwa et al. (2010) [27]	2008	1000 (5620)	Individual and Societal	General health	-	3(1)	KRW	68000000–79,000,000 (46,000,000)	72000000 (46000000)	103641 (66,644)	67360 (66,644)	High-income
	Song HJ et al. (2018) [39]	2015	507	Individual	General health	EQ-5D	4	KRW	15000000–35,000,000	24,750,000	31,078		
**Spain**	Pintto-Prades et al. (2009) [40]	N/A	892	Individual	General health	EQ5D, SG	37	EUR	4585–123,724	30,385	48,757	53888 (13,171)	High-income
	Robinson et al. (2013) [31]	2008	2679 (21896)	Individual	General health	EQ-5D, TTO, SG	8(8)	EUR	25629–52,876 (6671–12,669)	36780 (8,089)	59019 (13,171)		
Sweden	Robinson et al. (2013) [31]	2008	2604 (21896)	Individual	General health	EQ-5D, TTO, SG	8(8)	EUR	16908–35,200 (3235–7,842)	26769 (4,803)	39429 (6,985)	37033 (6,985)	High-income
	Sund et al. (2018) [41]	2014	1400	Individual	General health	EQ-5D	1	SEK	280,000	280,000	34,636		
Taiwan	Shiroiwa et al. (2010) [27]	2008	504 (5620)	Individual and Societal	General health	-	3(1)	NT$	1800000–2,100,000 (1,400,000)	1933333 (1400000)	140840 (55,973)	140840 (55,973)	High-income
Thailand	Thavorncharoensap et al. (2013) [42]	N/A	4320	Individual	General health	EQ-5D	16(16)	Baht	72238–350,161 (38396–150,376)	169841 (76,211)	15215 (6,827)	15938 (6,827)	Upper-middle-income
	Thavorncharoensap et al. (2013) [43]	2008	1191	Individual	Blindness, paraplegia and allergies	TTO, VAS	12	Baht	26000–285,000	109,500	10,908		
	Nimdet et al. (2015) [44]	2013	600	Individual	General health	EQ-5D	1	Baht	243,120	243,120	21,691		
UK	Shiroiwa et al. (2010) [28]	2008	1002 (5620)	Individual and Societal	General health	-	3(1)	GBP	23000–38,000 (12,000)	29000 (12000)	49193 (20,621)	41723 (13,630)	High-income
	Robinson et al. (2013) [31]	2008	2312 (21896)	Individual	General health	EQ-5D, TTO, SG	8(8)	EUR	13228–29,308 (3064–6,775)	20193 (3,959)	34253 (6,639)		
USA	Byrne et al. (2005) [45]	2001	193	Individual	Osteoarthritis and general health	SG, TTO	6	USD	2844.1–5,690	3,981	5,750	46265 (26,259)	High-income
	Lieu et al. (2009) [46]	2005	478	Individual	Shingles and hepatic neuralgia	TTO	1(1)	USD	33000 (12,000)	33000 (12000)	43198 (15,708)		
	Shiroiwa et al. (2010) [28]	2008	1000 (56200)	Individual and Societal	General health	-	3(1)	USD	62000–96,000 (31,000)	75667 (31000)	89848 (36,810)		

TTO, Time-trade off; SG, Standard gamble.

**Fig 2:**
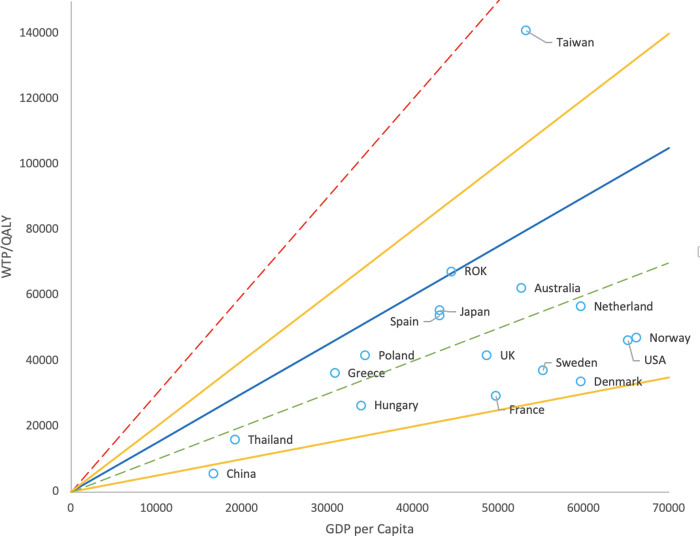
GDP per capita plotted against mean WTP/QALY by country. Red line: 3 times GDP per capita. Green line: Equal to GDP per capita. Yellow line: 0.5 (upper) or 2 times (lower) GDP per capita. Blue line: 1.5 times GDP per capita. WTP/QALY is within the range of 0.5 to 2 times GDP per capita for 88.2% (15/17) of countries. WTP/QALY is within the range of 0.5 to 1.5 times GDP per capita for 82.4% (14/17) of countries.

**Fig 3:**
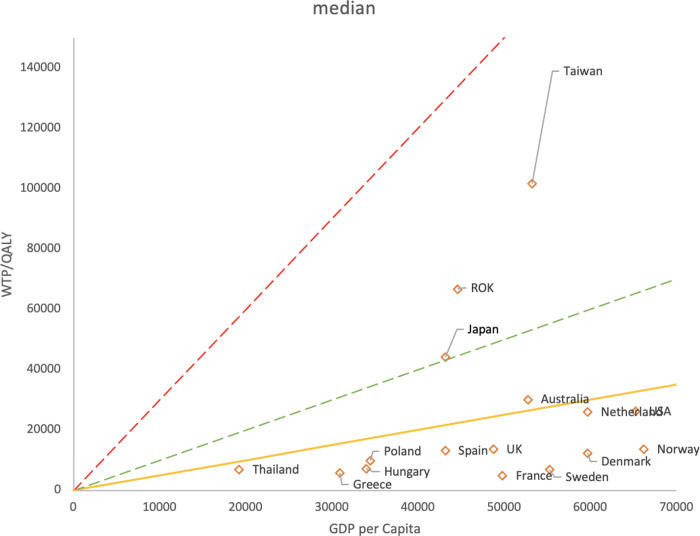
GDP per capita plotted against median WTP/QALY median by country. Red line: 3 times GDP per capita. Green line: Equal to GDP per capita. Yellow line: 0.5 times GDP per capita. WTP/QALY is less than 0.5 times GDP per capita for 75% (12/16) of the countries.

Comparison of the mean WTP/QALY with GDP per capita shows that 41.2% (7/17) of the countries were located in the WHO-recommended range of 1 to 3 times GDP per capita (red line). When the criterion was set to 0.5 to 2 times GDP per capita (yellow line), fifteen out of seventeen countries (88.2%) were located in the range (Fig 2). Within 0.5 to 1.5 times GDP per capita, fourteen out of seventeen countries (82.4%) were located in the range. As regards the median, in twelve out of sixteen countries (75%), the values were distributed below 0.5 times GDP per capita (Fig 3).

**Fig 4:**
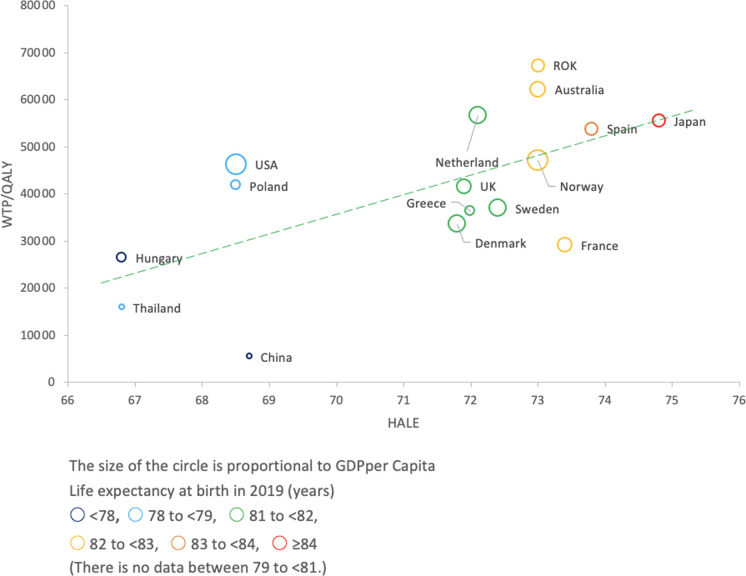
GDP per capita plotted against WTP/QALY by country. Taiwan was excluded as an outlier. Pearson’s correlation analysis was performed, and a statistically significant correlation was found (r = 0.636, p = 0.008).

We found no correlation between WTP/QALY and HALE (r = 0.246). Cook’s distance and DFFITS were calculated, and we confirmed that Taiwan was an outlier (detailed data and code are available in Supporting information (S2 Table, S2 File)). When Taiwan was excluded, a statistically significant positive correlation was observed (Fig 4, r = 0.636, p = 0.008).

## Discussion

To make effective use of medical resources, policymakers in each country need to have a policy regarding their proper allocation. Cost-effectiveness evaluation is necessary for decision-making. In this study, we examined GDP per capita and HALE as indicators of the CE threshold. A comparison of the average WTP/QALY with GDP per capita showed that the values of WTP/QALY for most countries were located in the range of 0.5–1.5 times GDP per capita.

According to the WHO-recommended threshold, less than the GDP per capita is rated as "highly effective" and from one to three times GDP per capita as "cost-effective" [4]. The former assessment is based on the assumption that if an intervention can produce one QALY per year at the cost of less than the GDP per capita, the resulting value-added will exceed the cost of the investment [4,47].

In this study, the mean values of WTP/QALYs were distributed well below the WHO upper threshold (Fig 2). Therefore, setting a CE threshold based on the WHO criteria may give the impression of being relatively expensive to the general population. Moreover, expensive new drugs are being launched rapidly, and thus the cost of gaining a new QALY in developed countries might be increasing. In this case, the cost of gaining additional health is likely to be higher than the WTP that people anticipate. Especially in health care, there is a large asymmetry of information, which may lead to a discrepancy between the actual threshold and the WTP.

Countries in the upper-middle-income group tend to have lower WTP/QALYs than those in the high-income group. When healthcare expenditure is low, the law of diminishing marginal utility suggests that the health value gained may be relatively high in relation to the cost. In such countries, the criterion of 0.5–1.5 times GDP shown in this study may be potentially suitable. If ⊿Cost/⊿QALY is less than the GDP per capita, the cost-effectiveness is excellent. However, if the CE threshold is set to less than the GDP per capita, the use of health technologies that theoretically have a higher return than the cost could be restricted. Therefore, an actual CE threshold of 1–1.5 times GDP per capita is considered to be an appropriate range. On the other hand, there is an example of a threshold of less than the GDP per capita. In Thailand, a threshold of 0.8 times GDP (100,000 baht) was set in 2007 when the list of essential medicines was created, and this threshold is used for price adjustment [4,43]. This criterion was set based on gross national income (GNI), and was not intentionally set below the GDP per capita [48]. This policy led to price reductions of 72% for tenofovir and 69% for oxaliplatin in Thailand [49]. The prices after these reductions are about the same as in Japan, but are relatively high considering the GDP ratio. This suggests that the cost before the price reduction was high, so the price adjustment based on the threshold may have worked appropriately [50,51].

In the comparison of median WTP/QALYs and GDP per capita, most values were in the range of 0.5 times or less. Furthermore, the median value was lower than the mean value in all countries. This result is similar to that reported by Song et al (2018) and implies that the distribution of WTP is skewed with a long tail to the right [39]. Several previous studies have reported that WTP has larger values when people’s income is higher. Considering that the distribution of WTP has a similar pattern to that of income, the median value is expected to be lower than the mean [4,28]. The median value would be useful in situations where the burden of medical expenses on individuals is large. In contrast, from the government’s perspective, the impact of the total cost on the budget is important. Therefore, the mean value is more important than the median value in a state where the burden on individuals is reduced by insurance.

Several values of median WTP/QALY are scattered above the 0.5 times line in Fig 3. One possible reason for this is that fewer values of the median were available. That is, the distribution on the lower side (points distributed below 0.5 times) is strongly influenced by the article [31], while the distribution on the upper side contains data extracted from the article [28]. In addition, a previous study has shown that "median values were (predominantly) independent of the size of the utility gain." [38]. Currently, no clear methodology for determining WTP/QALYs has been established, and the median may not be reliable as a basis for real-world practice.

In both the mean and median graphs, the variance of WTP/QALY became larger as GDP per capita increased. This can be expected, as the range of values presented as WTP widens as surplus wealth increases with the growth of GDP.

In this study, only Taiwan was located away from and on the upper side. This is similar to the result in the survey by Shiroiwa et al [28], but the reason for this trend is not clear. Shiroiwa et al (2010) investigated the WTP from the perspective of individual, society, and family, and the results showed that in Western countries, the order of WTP was family > society > individual; in Asian countries, the order was society > family > individual; and in Taiwan, the order was individual > family > society, with the WTP for individuals having the highest value [28]. This unique preference in Taiwan may have affected the present study as well. Furthermore, Taiwan’s implied PPP LCU per USD is about 13, whereas the exchange rate deviates significantly to about 28. This divergence may have affected our results, because PPP and exchange rate rarely diverge significantly.

In the comparison between WTP/QALY and HALE, a statistically significant positive correlation (r = 0.638 p = 0.008) was found when Taiwan was excluded. WTP is correlated with expenditure. Therefore, it can be inferred that when WTP is high, the expenditure on health increases, and this is reflected in HALE. This behavior is in accordance with the Grossman model of the relationship between health and consumption [52]. In addition, since national income level and other factors are considered in setting the threshold, it can be inferred that there is a correlation between CE threshold and HALE.

However, even if a high threshold is set, it is unlikely that the effect will be quickly reflected in healthy life expectancy. In addition, setting unnecessarily high thresholds becomes expensive, and might lead to the collapse of the medical insurance system. This could be avoided by increasing the individual burden and reducing the payers’ burden, but this would result in worsening access to health care resources, hastening the loss of health capital. In many countries, the price of medical care can be adjusted publicly. Therefore, the government can decrease health care costs by lowering prices, but this can be expected to lead to a decline in the quality of services and a delay in research and development of new drugs and new medical devices. However, in Japan Shigeoka et al [53] found that, although price elasticity was reduced by increasing co-payments, this did not affect health. Thus, if out-of-pocket costs are low, it seems likely that costs are excessive and increasing patient out-of-pocket costs could be an effective policy.

The range of estimated WTP/QALYs in the present study can be used as a guide for threshold setting even in countries where no survey of WTP has been conducted. Nevertheless, each country has its own unique circumstances, and thus it is desirable to consider a range of evidence when setting the threshold. The current, widely known WHO criteria seem high when compared to the results of this study. However, greater spending usually means that more medical technology becomes available. Even spending a large amount of money is expected to improve people’s health and advance the development of medical technology. For this reason, we think that the threshold criteria currently being applied should not be lowered.

This study has several limitations. First is the paucity of available data: not only are there rather few countries in which WTP/QALY surveys have been conducted, but also there is a large variation in the number of surveys across countries. As with many measures, different calculation methods will give different values for WTP/QALYs. It should be possible to reduce the variability by using multiple WTP/QALY values calculated using validated methods and obtaining an average value. Second, factors related to healthy life expectancy include behavior-related factors such as medical visits and treatment and passive factors such as sanitation. Increasing health capital through consumption behavior can be expected to be related to WTP, but the extent to which passive factors are related to WTP is unclear. WTP is correlated with income, and countries with higher income levels can build more advanced infrastructure. However, it cannot be said that there is a direct relationship with WTP, so it is necessary to investigate this issue in the future. Furthermore, the WTP/QALYs in this study are based on the preferences of the general population. They can be used as a reference when setting the threshold, but it should be noted that the relevance to the actual threshold that is set remains uncertain.

In addition, since this survey used only English literature and limited databases, there may be a publication bias. There are also other possible biases due to the scenario in each survey. Previous studies have shown that scenarios can affect WTP/QALY values [8]. Four studies used specific disease scenarios (China, Thailand, USA) [29,43,45,46]. Among them, the Thailand and US studies present lower values. Such studies may tend to have lower values than studies that do not assume a specific disease, such as EQ-5D.

## Conclusion

Our results suggest that 0.5–1.5 times GDP per capita is appropriate as a threshold setting range for WTP/QALYs. This range is lower than the WHO range (1 to 3 times GDP per capita), but it could be used to set the CE threshold based on consumer preferences. We found a correlation between WTP/QALYs and HALE, which suggests that investment in health is reflected in the outcomes. In the future, as more WTP/QALYs are calculated around the world, this kind of analysis may provide clearer insights for setting policy.

## Supporting information

S1 Checklist (DOCX)

S2 Checklist (DOCX)

S1 Fig (TIFF)

S2 Fig (PNG)

S1 Table (XLSX)

S2 Table (XLSX)

S1 File (DOCX)

S2 File (IPYNB)
