# Supplementary figures and images for: Estimating the range of incremental cost-effectiveness thresholds for healthcare based on willingness to pay and GDP per capita: A systematic review
Source: PLoS One. 2022 Apr 14;17(4):e0266934. doi: 10.1371/journal.pone.0266934 (PMC9009631; doi:10.1371/journal.pone.0266934)

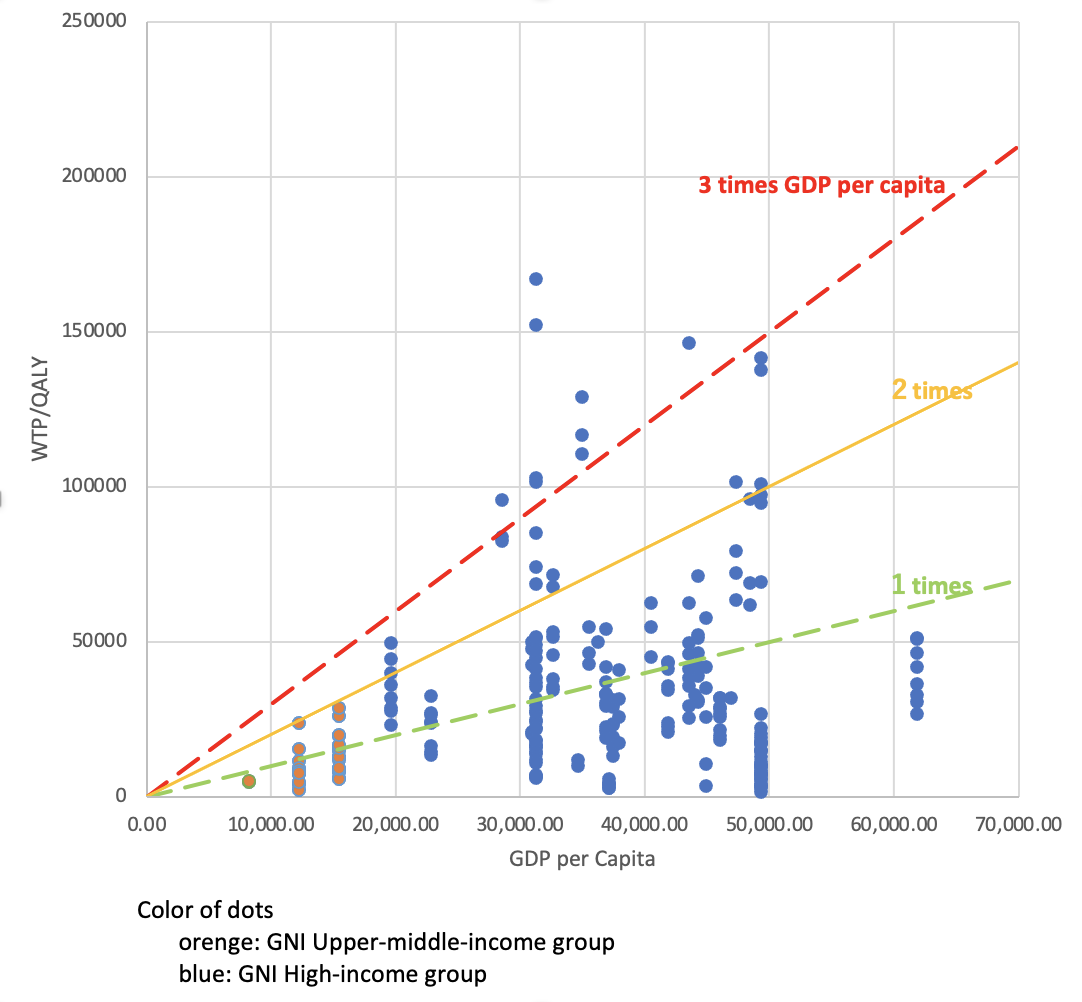

Supplement: S1 Fig — (TIFF) [file pone.0266934.s003.tiff]

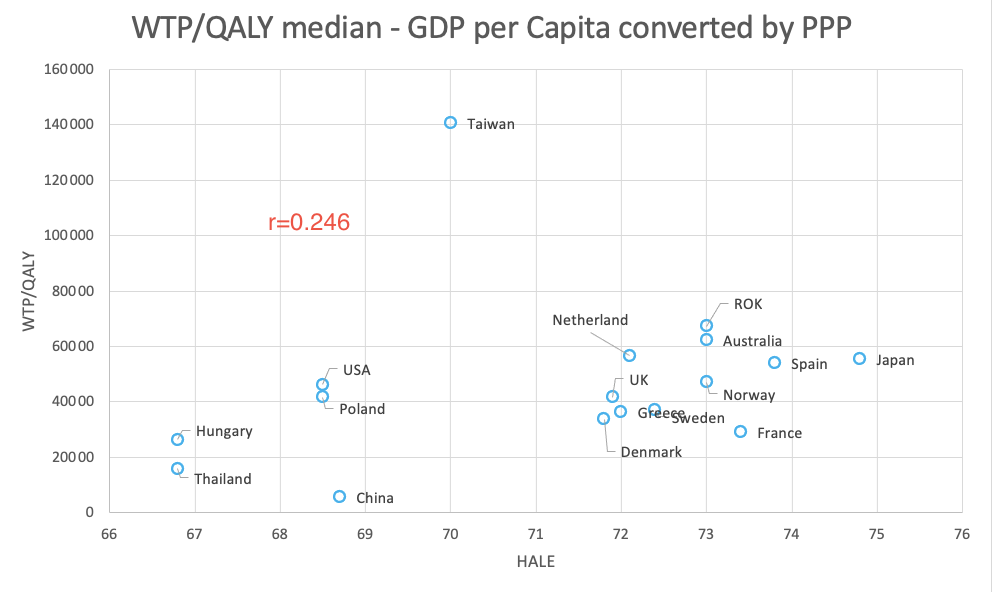

Supplement: S2 Fig — (PNG) [file pone.0266934.s004.png]
