# Supplementary material for: Estimating the range of incremental cost-effectiveness thresholds for healthcare based on willingness to pay and GDP per capita: A systematic review
Source: PLoS One. 2022 Apr 14;17(4):e0266934. doi: 10.1371/journal.pone.0266934 (PMC9009631; doi:10.1371/journal.pone.0266934)
Supplement: S1 File — (DOCX) [file pone.0266934.s007.docx]

S3. Detail of search strategy.

| Database searched | Timespan | Search strategy |
| --- | --- | --- |
| MEDLINE | 2000/01/01 - 2020/12/31 Publication Date | #1. "quality adjusted life year" (6002) #2. "quality adjusted life years" (18393) #3. "disability adjusted life year" (887) #4. "disability adjusted life years" (3431) #5. "cost benefit analysis"[Mesh] (89696) #6. "willingness to pay" (7005) #7. #1 OR #2 OR #3 OR #4 (23494) #8. #5 AND #6 AND #7 (2394) #9. #8 AND ("2000/01/01"[ Date - Publication] : "2020/12/31"[Date - Publication]) (2176) |
| EMBASE | 2000/01/01 - 2020/12/31 Publication Date | #1. "quality adjusted life year" (32418) #2. "quality adjusted life years" (3490) #3. "disability adjusted life year" (14971) #4. "disability adjusted life years" (4086) #5. "cost benefit analysis"[Mesh] (93901) #6. "willingness to pay" (11114) #7. #1 OR #2 OR #3 OR #4 (38670) #8. #5 AND #6 AND #7 (666) #9. #8 AND ("2000/01/01"[ Date - Publication] : "2020/12/31"[Date - Publication]) (587) |
| Web of Science | 2000/01/01 - 2020/12/31 Publication Date | #1. TS=(“quality adjusted life year*”) (23,468) #2. TS=(“disability adjusted life year*”) (5,871) #3. TS=(cost benefit analysis) (188,934) #4. TS=(willingness to pay) (29,686) #5. #1 OR #2 (27,696) #6. #3 AND #4 AND #5 (2,846)  #6 AND (publication date=(1980/01/01-2020/12/31)) (2,502) |
